# Surveillance donor-specific antibody and pathologic antibody-mediated rejection testing in heart transplant patients in the contemporary era

**DOI:** 10.1016/j.healun.2025.01.019

**Published:** 2025-02-04

**Authors:** Vincenzo Cusi, Ashley Cardenas, Yuko Tada, Florin Vaida, Nicholas Wettersten, Jennifer Chak, Victor Pretorius, Marcus Anthony Urey, Gerald P. Morris, Grace Lin, Paul J. Kim

**Affiliations:** a Department of Medicine, University of California San Diego Health, San Diego, California;; b Department of Pathology, University of California, San Diego, California;; c Department of Family Medicine and Public Health, UC San Diego, La Jolla, California;; d Cardiology Section, Veterans Affairs San Diego Healthcare System, San Diego, California;; e Division of Cardiovascular and Thoracic Surgery, Department of Surgery, University of California, San Diego, California.

**Keywords:** antibody-mediated rejection, de novo donor-specific antibodies, heart transplantation, cardiac allograft dysfunction, HLA antibody, primary graft dysfunction

## Abstract

**BACKGROUND:**

Surveillance donor-specific antibody (DSA) and pathologic antibody-mediated rejection (pAMR) testing is recommended in the first year after heart transplantation (HTx) in adult patients. Whether pAMR testing adds prognostic information to contemporary DSA testing has not been fully studied.

**METHODS:**

This was a single-center study of consecutive endomyocardial biopsies (EMB) performed between November 2010 and February 2023 in adult HTx patients. The primary objective was to evaluate whether pAMR testing contributes additional information to DSA testing to better predict overall survival. Secondary end-points included cardiac survival and allograft dysfunction.

**RESULTS:**

A total of 6,033 EMBs from 544 HTx patients were reviewed for the study. The pAMR +/DSA+ patients had significantly lower overall (p_c_ = 0.013) and cardiac survival (p_c_ = 0.002), while the pAMR+/DSA− and pAMR−/DSA+ patients showed no difference in either outcome compared to the pAMR−/DSA− group. We found significantly lower overall survival in pAMR+/DSA+ patients with allograft dysfunction (p_c_ < 0.001) but not in pAMR+/DSA+ patients without allograft dysfunction (p_c_ = 0.569), when compared to the pAMR−/DSA− without allograft dysfunction group. The pAMR +/DSA+ patients with cardiac allograft dysfunction accounted for 18% of deaths or cardiac retransplants while only representing 4% of the HTx cohort. Moderate or severe primary graft dysfunction (PGD) also was a novel risk factor for the development of de novo DSAs (dnDSA) by 4 weeks post-HTx (*p* = 0.025).

**CONCLUSIONS:**

Surveillance DSA testing may effectively identify high-risk pAMR+ patients. Earlier DSA testing at 10 to 14 days post-HTx should also be considered in moderate or severe PGD patients. J Heart Lung Transplant

## Background

Recognition and standardization for the diagnosis of pathologic antibody-mediated rejection (pAMR) occurred in 2013 by the International Society for Heart and Lung Transplantation (ISHLT) working group, where surveillance for pAMR in adult heart transplantation (HTx) patients was first recommended.^1^ With the goal of making pAMR a pathologic diagnosis, akin to acute cellular rejection,^2^ the ISHLT working group also removed the presence of donor-specific antibody (DSA) and cardiac allograft dysfunction for pAMR diagnosis. These pivotal changes were also made to address the concern for underdetection of asymptomatic pAMR.

While DSA and pAMR testing continue to be recommended together after HTx,^3^ how both results should influence the management of HTx patients remains unclear.^4^ In this single-center study, we aimed to evaluate whether pAMR testing provides additional information to DSA testing to predict overall survival in HTx patients. In addition, because HTx population demographics and HTx management have significantly changed over time,^5^ we performed a comprehensive analysis to identify potential predictors for de novo DSAs (dnDSA), pAMR, cardiac allograft dysfunction, cardiac allograft vasculopathy (CAV), cardiac and overall survival in patients with pAMR and DSA testing for the contemporary era (2010-current).

## Methods

### Data sharing

The data that support the findings of this study are openly available in Mendeley Data (doi:10.17632/d4f7g8hs5z.3).

### Study design

Consecutive patients who were 18 years of age or older and underwent HTx between November 2010 to February 2023 were retrospectively reviewed. Patients without prior pAMR and DSA results available were excluded. Database lock occurred March 2024, 1 year after the inclusion of the final patient. The endomyocardial biopsies (EMB) surveillance protocol followed for all HTx patients at the University of California, San Diego Health (UCSD) during the study period included C4d immunofluorescence at 10 to 14 days, 1 month, 3 months, 6 months, and 12 months during the first year post-HTx.^1,6^ Surveillance DSA testing is performed at the same time intervals during the first year post-HTx and also quarterly after the first year post-HTx.^3^ C4d immunofluorescence and DSA testing are also performed anytime there is a clinical suspicion of rejection, which includes signs or symptoms of congestive heart failure, echocardiographic evidence of graft dysfunction, new arrhythmias, or where a repeat EMB is requested to confirm the resolution of a recent episode of pAMR.^6^ VC, AC, PB, and JC collected patient data and clinical outcomes from the electronic medical record. Approval for this study was provided by the UCSD Office of IRB Administration (IRB #805675). This study adheres to the principles of the Declaration of Helsinki formulated by the World Medical Association, the Declaration of Istanbul, and the ISHLT statement on Transplant Ethics.

### Pathologic tissue exams and human leukocyte antigen (HLA) antibody testing

C4d immunofluorescence was performed starting November 2010 with positivity defined according to the ISHLT Working Formulation; however, contemporary ISHLT pAMR grading was implemented at UCSD in July 2015.^1^ Thus, EMB samples before July 2015 were regraded using the current pAMR grading scheme for this study (GL). Anti-HLA testing was performed using single-antigen bead LABScreen HLA Class I and II assays (One Lambda, Canoga Park, CA) on LabScan 100 and FlexMap 3D (Luminex, Austin, TX) instruments. Data were analyzed using HLA Fusion software (One Lambda). Antibodies with normalized mean fluorescence intensity (MFI) values > 3,000 were identified as positive, based upon the likelihood of causing a positive flow cytometric crossmatch.^7^ DSA were identified by comparison of antibody testing results to donor HLA typing. Concurrent DSA positivity was defined as occurring within a month of a pAMR diagnosis.

### Clinical outcomes and variables

The primary outcome was freedom from all-cause death or cardiac retransplant, that is, overall survival. The cause of death was also adjudicated by 3 experienced cardiologists (NW, YT, PJK). Secondary outcomes evaluated were as follows: freedom from cardiac allograft failure (also known as cardiac survival), future episodes of pAMR or dnDSA detection, concurrent or future cardiac allograft dysfunction (echocardiogram demonstrating left ventricular ejection fraction < 50%)^8,9^ occurring after 1-week post-HTx, and ISHLT CAV grade 2 or greater.^10^ Moderate or severe primary graft dysfunction (PGD) was diagnosed in the first 24 hours after HTx, using modified criteria from the ISHLT 2014 consensus conference.^11–13^ Specifically, moderate or severe PGD was defined by any of the following criteria: (1) mechanical support with need for right ventricular assist device, (2) mechanical support with left ventricular assist device implantation or venoarterial extracorporeal membrane oxygenation, or (3) left ventricular ejection fraction 45% or less and need for (1) intra-aortic balloon pump or (2) high-dose inotropic support defined as an inotrope score > 10 (Inotrope score = dopamine + dobutamine + amrinone + milrinone × 15 + epinephrine × 100 + norepinephrine × 100 with each drug dosed in mcg/kg/min).^11^ Hemodynamic criteria were not used as many identified PGD patients had tenuous intraoperative courses with inconsistent charting of hemodynamic variables in this setting.^12,13^ For this study, moderate or severe PGD patients were evaluated as prior literature suggests these 2 categories are the most clinically relevant.^14^ Documentation by a clinical team member of immunosuppressive medication nonadherence after HTx was recorded retrospectively by medical chart review (VC, AC, PB, JC).^15–17^

### Statistical analysis

Demographic and clinical variables were analyzed with standard statistics as previously described for continuous and count variables.^6^ The association of pAMR/DSA groups with time-to-event outcomes was evaluated using single predictor and multipredictor Cox proportional hazards models. The multipredictor models for overall and cardiac survival were adjusted for recipient age, sex, and race/ethnicity. Additional exploratory analyses investigated factors associated with time-to-event outcomes using Cox models applying a backward model selection procedure with *p*-value ≤ 0.15 threshold for inclusion. Due to the large number of potential predictors, the starting model only included predictors with *p* ≤ 0.15 in the single predictor analyses. Collinearity was evaluated by calculating the variance inflation factor for each independent variable, and a cutoff of 2 was used to drop a variable. Cox models with time-dependent covariates were used when the proportional hazards assumption of constant hazard ratios was violated. Sensitivity analyses were performed with the pAMR/DSA class within the same patient as a time-dependent covariate in Cox models for the outcomes of overall or cardiac survival, to allow for different risks for different pAMR/DSA classes throughout the follow-up. To adjust for different causes of death as competing outcomes, a competing-risk regression model was constructed using the Fine and Gray method. We implemented bootstrapping, repeated 10,000 times, to generate approximate sampling distributions that provide the mean and 95% confidence intervals for the positive predictive values for pAMR diagnosis using different dnDSA patterns. The *p*-value of the comparison between dnDSA groups is based on the tail probability of the value of 0 (no difference) in the bootstrap distribution of the difference statistic, multiplied by 2 (2-tailed test).

Analysis was conducted in R (R Core Team, 2024). We used the Bonferroni-Holm procedure whenever multiple comparisons were performed while implementing a particular statistical hypothesis test. The corrected *p*-values are designated as p_c_. For single hypothesis testing, we report the uncorrected *p*-value. *P* or p_c_ < 0.05 is considered significant.

## Results

### Patient demographics

**Figure 1:**
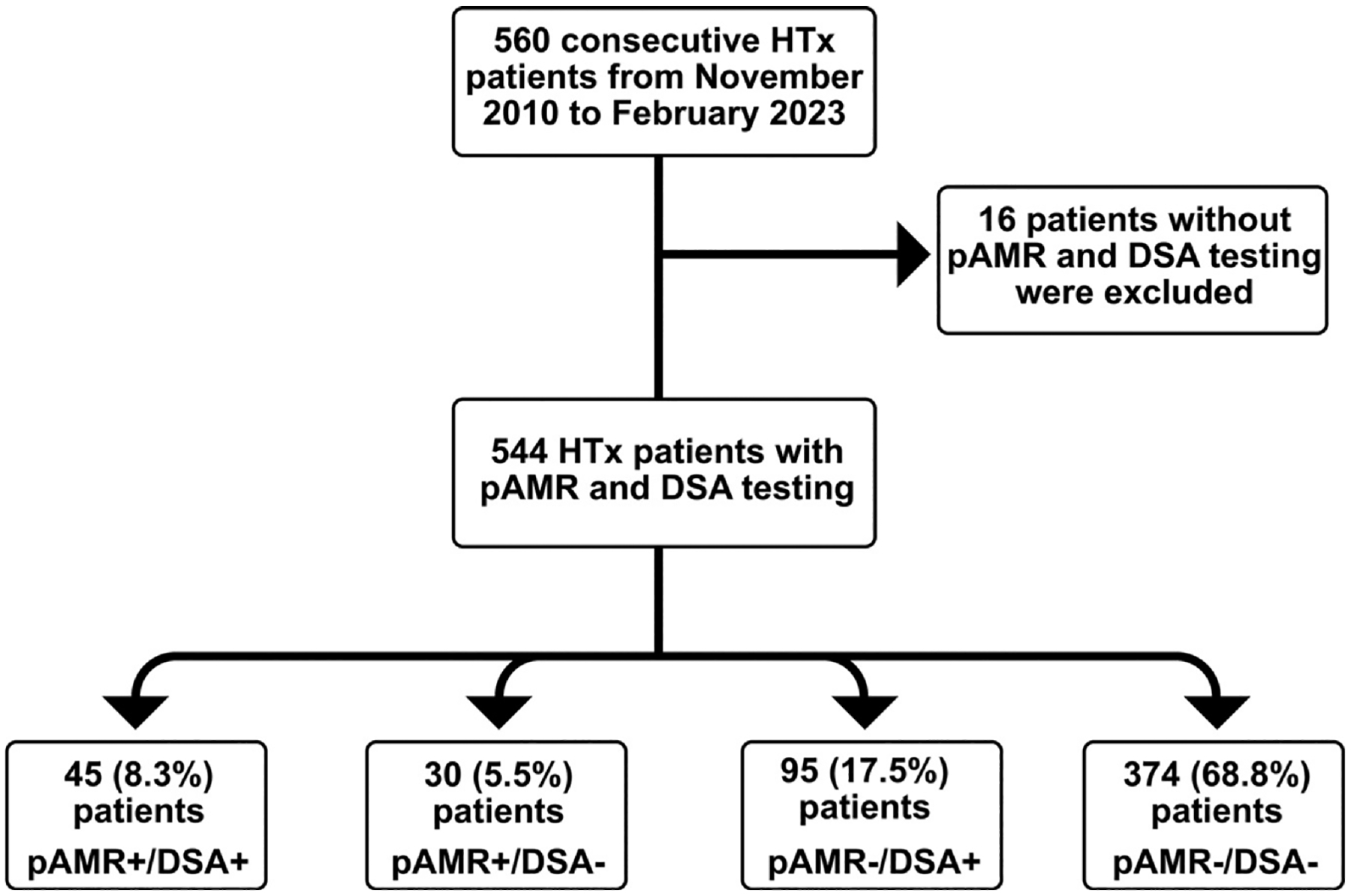
Flow diagram for heart transplants included then grouped based on history of pAMR and DSA results. DSA, donor-specific antibodies; HTx, heart transplantation; pAMR, pathologic antibody-mediated rejection.

A total of 6,033 EMBs from 544 HTx patients, including 4 cardiac retransplants, were evaluated (Figure 1). We divided all patients into 1 of 4 groups based on the history of pAMR and DSA results: pAMR+/DSA+ (*n* = 45, 8.3%), pAMR+/DSA− (*n* = 30, 5.5%), pAMR−/DSA+ (*n* = 95, 17.5%), and pAMR−/DSA− (*n* = 374, 68.8%) patients. In our prespecified analysis, we considered each HTx to be classified into 1 group, based on our hypothesis that the primary outcome of death or retransplant would be associated with the risk of the “highest” pAMR/DSA class achieved post-HTx. Thus, we considered the highest pAMR/DSA class to be pAMR+/DSA+, intermediate classes to be pAMR+/DSA− or pAMR−/DSA+, and the control group to be pAMR−/DSA−.

Characteristics of the study population are summarized in Table 1 and Table S1. HTx recipients were followed for a total of 1,999.2 person-years from the time of HTx.

### Association of pAMR/DSA classification with overall and cardiac survival

A total of 61 (11.2%) patients died or underwent cardiac retransplant during the follow-up period. Adjudicated causes of death are provided in Table S2. Initial adjudication of cause of death agreed 87.7% of the time with a Cohen’s kappa of 0.82 (0.69, 0.94; *p* < 0.001).

**Figure 2:**
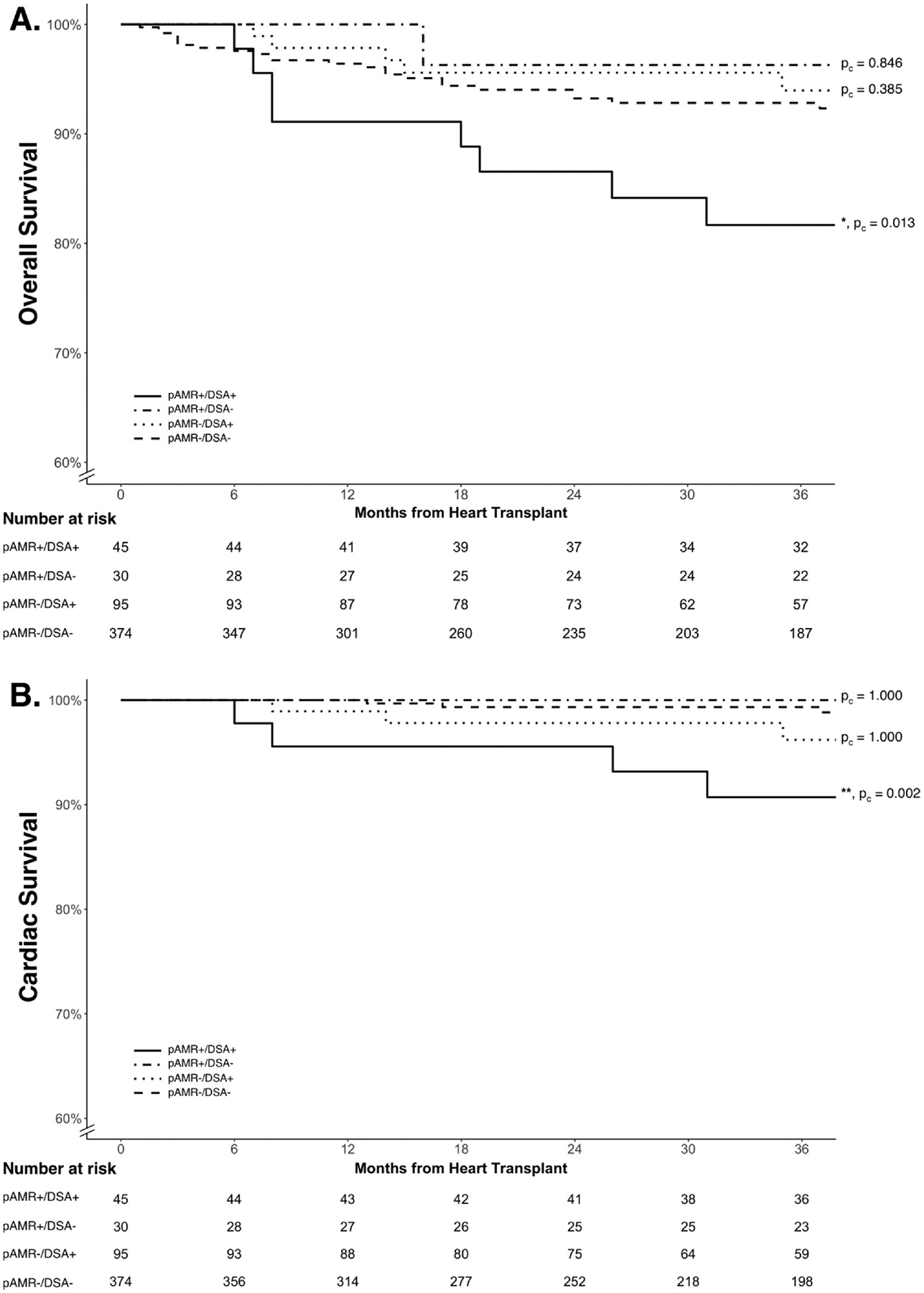
Kaplan-Meier curves by pAMR/DSA groups for (A) overall and (B) cardiac survival. The pAMR+/DSA+ patients show significantly reduced overall and cardiac survival compared to the pAMR−/DSA− group. The pAMR+/DSA− and pAMR−/DSA+ groups were not significantly different from the pAMR−/DSA− group. Fine-gray subdistribution hazard model was used to account for competing causes of death. Adjusted *p*-values for pairwise comparisons compared to the pAMR−/DSA− reference class are provided next to the survival curves. DSA, donor-specific antibodies; pAMR, pathologic antibody-mediated rejection. *p < 0.05; **p < 0.01.

We found that pAMR+/DSA+ patients had significantly lower overall (hazard ratio = 2.63; 95% confidence interval [CI], 1.35–5.11; p_c_ = 0.013; Figure 2A) and cardiac survival (hazard ratio = 7.00; 95% CI, 2.31–21.20; p_c_ = 0.002; Figure 2B) while the pAMR+/DSA− and pAMR−/DSA+ patients showed no difference in either outcome (p_c_ = 1.000) compared to the pAMR−/DSA− group. Sensitivity analyses performed to account for changes in pAMR/DSA classes within the same patient also corroborated our initial findings for overall and cardiac survival (Tables S3 and S4). Additionally, sensitivity analyses excluding HTx patients with only preformed DSAs demonstrated significantly lower overall (p_c_ = 0.025) and cardiac survival (p_c_ = 0.002) in pAMR+/DSA+ patients while pAMR+/DSA− and pAMR−/DSA+ patients again showed no difference in either outcome (p_c_ = 0.941) compared to pAMR−/DSA− patients.

### Analysis of pAMR+/DSA+ patients

The pAMR+/DSA+ patients demonstrated significantly later diagnosis of pAMR after HTx compared to the pAMR +/DSA− patients (33.6 vs 3.7 weeks; *p* = 0.004). The majority (62.2%) of pAMR+/DSA+ patients were diagnosed by pAMR+ and DSA+ results concurrently, that is, within 1 month of either test. A small number of patients (6.7%) were found to have a DSA+ result 3 months after a pAMR+ result. No patient with an initial pAMR+/DSA− classification had a primary outcome of death or retransplant within a year of the pAMR+ result.

There was no significant difference in time to dnDSA positivity in pAMR+/DSA+ compared to pAMR−/DSA+ patients (20.3 vs 17.0 weeks; *p* = 0.941). Detection of both class I and II dnDSAs on initial DSA+ testing demonstrated the highest positive predictive value than other DSAs, as summarized in Table S5. Detection of both class I and II dnDSAs on initial DSA+ testing was also associated with greater odds for pAMR2 or pAMR3 grades than class II dnDSAs alone (Odds ratio = 5.92; 95% CI, 1.02–33.74; p_c_ = 0.025) and showed a trend for greater odds compared to class I dnDSAs alone (Odds ratio = 4.67; 95% CI, 0.70–36.86; p_c_ = 0.147). Detection of both class I and II dnDSAs at any time post-HTx had a significantly higher risk of pAMR, cardiac allograft dysfunction, CAV, cardiac allograft failure, and all-cause death or retransplant (*p* < 0.001 for all). Class II dnDSAs alone (*p* < 0.001) predicted pAMR but no other clinical outcomes. We did not find specific class II dnDSAs alone that significantly increased the risk for pAMR compared to other class II dnDSAs. Class I dnDSAs alone did not predict pAMR or other clinical outcomes. Donor-derived cell-free (dd-cfDNA) DNA testing was also performed in 8 pAMR+/DSA+ patients within 14 days before a pAMR+ diagnosis. In all 8 patients, the dd-cfDNA fraction was greater than or equal to 0.20%.^9^

### Cardiac allograft dysfunction in pAMR+/DSA+ patients

We found that a pAMR+/DSA+ status independently predicted concurrent or future cardiac allograft dysfunction (Table 2). In contrast, the rates of cardiac allograft dysfunction for pAMR+/DSA− (*p* = 0.601) and pAMR−/DSA+ groups (*p* = 0.235) were not significantly different from the pAMR−/DSA− patients; these 3 groups demonstrated an occurrence of cardiac allograft dysfunction within the range of 10% to 16%. In pAMR+/DSA+ patients, diagnosis of pAMR in the first year or after 1-year post-HTx also did not show differences in cardiac allograft dysfunction, cardiac survival, or overall survival (data not shown).

**Figure 3:**
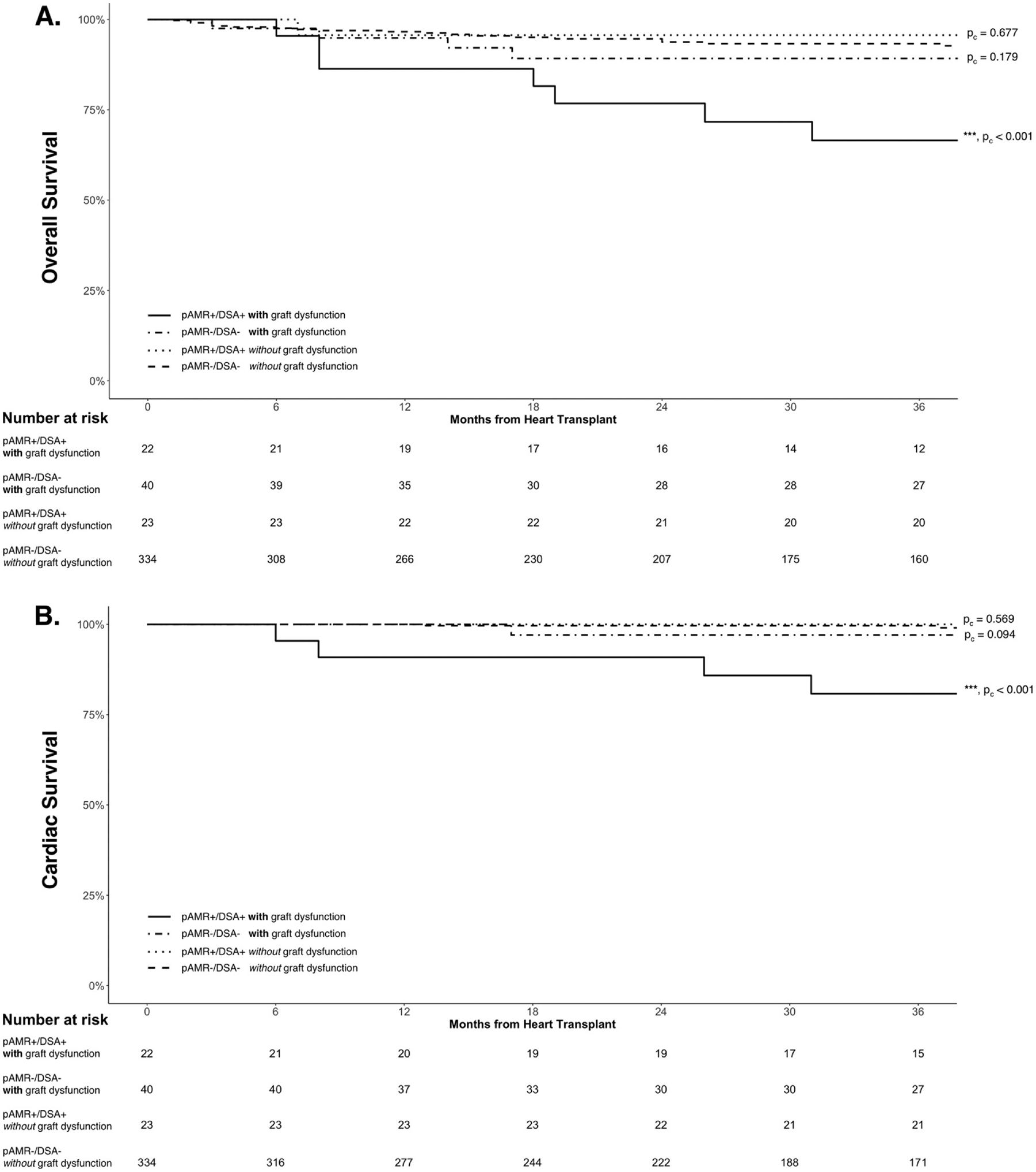
Kaplan-Meier curves by cardiac allograft dysfunction for (A) overall and (B) cardiac survival. There was significantly worse overall and cardiac survival in pAMR+/DSA+ patients with allograft dysfunction but not in pAMR+/DSA+ patients without allograft dysfunction, when compared to the pAMR−/DSA− without allograft dysfunction group. Adjusted *p*-values for pairwise comparisons compared to the pAMR−/DSA− patients without allograft dysfunction are provided next to the survival curves. ***p < 0.001. DSA, donor-specific antibodies; pAMR, pathologic antibody-mediated rejection.

Presence of cardiac allograft dysfunction was associated with lower overall and cardiac survival (Tables 3 and S6). Significantly lower overall and cardiac survival was demonstrated in pAMR+/DSA+ patients with allograft dysfunction but not in pAMR+/DSA+ patients without allograft dysfunction, when compared to the pAMR−/DSA− without allograft dysfunction group (Figure 3).

### Predictors for dnDSAs

**Figure 4:**
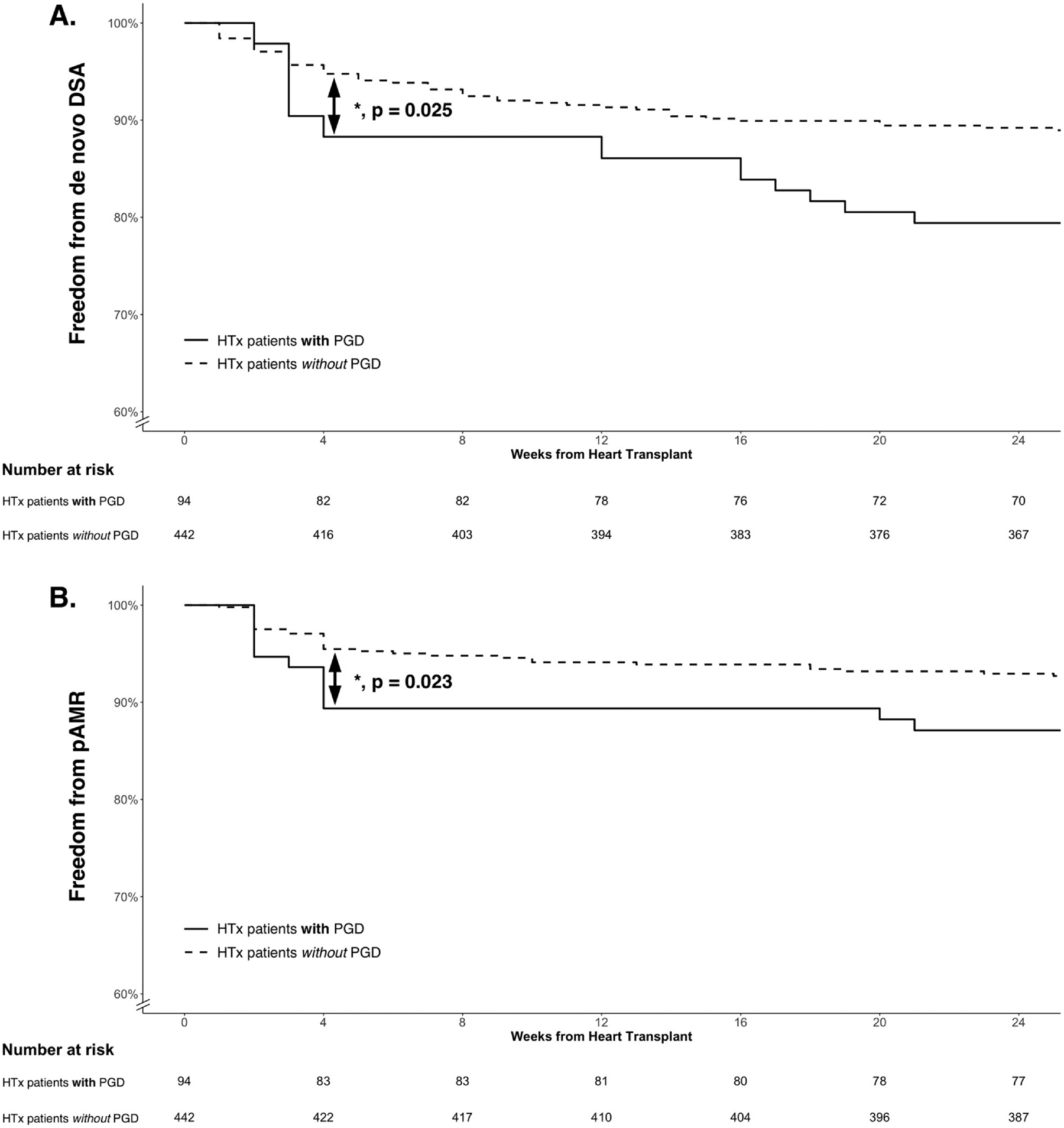
Kaplan-Meier curves by moderate or severe PGD for (A) freedom from de novo DSA detection and (B) freedom from pAMR. HTx patients with moderate or severe PGD demonstrated a significant increase in de novo DSAs and incidence of pAMR by 4 weeks post-HTx, after which the hazard ratios were not significantly different between HTx patients with and without PGD. *p < 0.05. DSA, donor-specific antibodies; HTx, heart transplantation; pAMR, pathologic antibody-mediated rejection; PGD, primary graft dysfunction.

Younger recipient age and medication nonadherence were independent predictors for development of dnDSAs (Table 4). However, we also observed that patients with moderate or severe PGD demonstrated a significant increase in dnDSAs and pAMR by 4 weeks post-HTx (Figures 4A and 4B). Donation after circulatory death (DCD) or utilization of venoarterial extracorporeal membrane oxygenation post-HTx was not associated with dnDSAs or pAMR.

## Discussion

In this retrospective cohort of 544 adult HTx patients with nearly 2,000 patient years of follow-up, we observed the following key findings. First, DSA testing with contemporary ISHLT pAMR grading identified pAMR+/DSA+ patients to have significantly lower overall and cardiac survival while pAMR+/DSA− and pAMR−/DSA+ patients showed no difference in either outcome compared to pAMR−/DSA− patients. Second, we found significantly lower overall and cardiac survival in pAMR+/DSA+ patients with cardiac allograft dysfunction but not in pAMR +/DSA+ patients without allograft dysfunction, when compared to the pAMR−/DSA− without allograft dysfunction group. Third, the detection of both class I and II dnDSAs had the highest predictive value for pAMR. Fourth, PGD is potentially a novel risk factor for the development of dnDSAs.

The current study findings suggest DSA testing may effectively identify high-risk pAMR+ patients. While Clerkin et al^18^ previously showed no difference in cardiac allograft failure in patients with pAMR or DSAs, substudy analysis showed that dnDSAs were associated with significantly lower cardiac survival, which are consistent with our findings as well as others.^19,20^ However, we go further to demonstrate that the risk of lower overall and cardiac survival is found specifically in the pAMR+/DSA+ group. Even though the relatively small number of events limited the statistical power for the pAMR+/DSA− and pAMR−/DSA+ groups, our findings suggest a viable strategy to reserve surveillance pAMR testing for DSA+ results. As a result, this strategy may reduce unnecessary pAMR testing.^6^ Coutance et al^8^ also recently proposed a clinical prediction model for pAMR, which includes history of a prior ISHLT pAMR2 diagnosis, cardiac allograft dysfunction, and DSA+ status as 3 of the 5 predictor variables. In contrast, our proposed strategy only requires DSA screening and may also provide an opportunity to prevent cardiac allograft dysfunction in pAMR+/DSA+ patients. Additionally, we note that new noninvasive biomarker testing, including dd-cfDNA, may play a future role in pAMR risk stratification. For instance, dd-cfDNA testing may be able to identify HTx patients earlier that are initially pAMR−/DSA+, before becoming pAMR+/DSA+ status.^21^ Ongoing multicenter observational studies (e.g., NCT03695601: SHORE) and randomized controlled trials (e.g., NCT06414603: ACES-EMB) may shed further light on the role of novel noninvasive biomarkers as an adjunct to DSA testing for further risk stratification of pAMR.

We hypothesize that ischemia-reperfusion tissue injury post-HTx predominantly contributes to early positive C4d immunostaining in pAMR+/DSA− patients, while complement activation due to dnDSAs is likely responsible for later positive C4d immunostaining that occurs in pAMR +/DSA+ patients. Previous studies have shown that positive C4d immunostaining in the early post-HTx period could result from the lectin complement pathway related to ischemia-reperfusion tissue injury.^22,23^ Mantell et al^24^ also have shown transcriptomic differences between the pAMR +/DSA+ and pAMR+/DSA− groups, with significant upregulation of genes related to immunity in the pAMR+/DSA+ patients. In addition, while non-HLA antibodies were not evaluated in this study and continue to be investigated in pAMR by others,^25^ there was no appreciable difference in outcomes in the pAMR+/DSA− compared to the pAMR−/DSA− group, even accounting for the possibility that non-HLA antibodies may be a contributing factor in some pAMR+/DSA− patients. However, given the relatively small number of events in our study, further research is needed to determine the role of non-HLA antibodies in pAMR.^26^

Furthermore, we found that the pAMR+/DSA+ patients with cardiac allograft dysfunction carried most of the increased risk for lower overall and cardiac survival and accounted for 18% of deaths or cardiac retransplants while representing only 4% of our HTx cohort. Cardiac allograft dysfunction was previously part of the clinical criteria for diagnosis of antibody-mediated rejection,^27^ and prior studies have also demonstrated the prognostic importance of cardiac allograft dysfunction, although not specifically in pAMR+/DSA+ patients.^28,29^ Thus, our study highlights the importance of surveillance for cardiac allograft dysfunction in pAMR+/DSA+ patients, given the significant increase in mortality once cardiac allograft dysfunction occurs.^30^ Of note, cardiac allograft dysfunction also occurred in pAMR−/DSA+ and pAMR−/DSA− patients, albeit at much lower rates. The cause was not identified in many cases (36%) and thus was considered to be nonspecific graft dysfunction.^31^

Detection of both class I and II dnDSAs increased the risk for pAMR+ diagnosis 3 times compared to the detection of other dnDSAs. In contrast to some other studies,^18,32^ we were not able to identify specific class II dnDSAs alone that significantly increased the risk for future pAMR more than other class II dnDSAs. We hypothesize some of the differences in literature are related to how HLA-DQ DSAs, the most frequently detected of the DSAs, are categorized when occurring in the presence of other DSAs.^33^ In the current study, we evaluated class I alone, class II alone, and both class I and II dnDSAs as separate categories and also evaluated for progression from one dnDSA category to another in subsequent testing.

Additionally, our study findings indirectly suggest increased immunogenicity associated with the detection of both class I and II dnDSAs compared to either class I or II dnDSAs alone. HTx patients with both class I and II dnDSAs demonstrated a higher rate of more severe initial pAMR+ grades compared to patients with either class I or II dnDSAs alone. Patients with both class I and II dnDSAs were also at a significantly increased risk for pAMR-related clinical outcomes, while patients with either class I or II dnDSAs alone were not. Prior studies have also shown that detection of class I and II DSAs, using the contemporary solid phase assays, were more predictive of persistent and cytotoxic DSAs than either class I or II DSAs alone.^34,35^ While class II DSAs can activate endothelial cells toward a proinflammatory response,^36^ the potential synergistic interaction of both class I and II DSAs warrants further study.

Finally, our data demonstrate moderate or severe PGD as a possible risk factor for early development of dnDSAs and pAMR, providing a novel insight into the potential relationship of PGD, dnDSAs, and pAMR in the peri-transplant period. Han et al^37^ previously showed a similar incidence of dnDSAs in patients with PGD compared to those without PGD. However, this study included a patient cohort with a much higher preformed DSA prevalence and a lower rate of moderate or severe PGD, which may explain the differences from our study findings. Additionally, at our institution, patients are initially tested for a DSA response at 10 to 14 days post-HTx. Thus, we found a significant increase in dnDSAs by 4 weeks post-HTx, suggesting a memory B-cell response related to PGD. Early inflammatory injury to the donor heart and increased transfusions of blood products,^38,39^ both factors associated with PGD, may contribute to allosensitization that leads to dnDSAs. Interestingly, DCD HTx was not associated with dnDSAs in a subgroup analysis, perhaps related to a prior observation that DCD HTx patients experience a different mechanism for PGD with quicker recovery than donation after brain death HTx patients.^13^ As the incidence for PGD continues at a high rate in the contemporary era,^40,41^ earlier DSA testing at 10 to 14 days post-HTx should be considered in moderate or severe PGD patients, and future studies should evaluate specific factors associated with PGD that may cause allosensitization.

### Study strengths and limitations

The strengths of this study include the following: its large size relative to similar studies of this nature in the contemporary landscape of HTx, the comprehensive analysis of multiple relevant copredictors, including medication nonadherence, for pAMR and DSA development, and the high proportion of dnDSAs in this HTx population. As our institution has historically limited HTx with a positive virtual crossmatch with a potential donor, this provided us the unique opportunity to study the association of dnDSAs with clinical outcomes. While this allowed for detailed analysis of dnDSAs, the small number of HTx patients with preformed DSAs also limited our observations of preformed DSAs with clinical outcomes. In addition, the relatively small number of events for the pAMR+/DSA− and pAMR−/DSA+ groups limited statistical power, and future studies from other centers should seek to confirm our findings for these groups. Our findings also do not provide guidance for treatment decisions, due to the wide variability in treatment regimens in our study. However, only a minority of pAMR +/DSA− (23%) and pAMR−/DSA+ (11%) patients were treated, and despite this, both groups had more favorable outcomes when compared to the pAMR+/DSA+ group (74% treated). Our center also utilizes C4d immunofluorescence with the use of CD68 and C4d immunoperoxidase staining in equivocal cases or when immunofluorescence is not feasible.^1^ Previous studies have shown that immunofluorescence and immunoperoxidase staining are similarly sensitive and specific for C4d positivity and our prevalence of pAMR was similar to prior studies.^18,42^ Additionally, as the median follow-up time was 3.1 years, our study was limited in the evaluation of CAV, which is considered a long-term post-HTx complication and has been described to be associated with pAMR.^3^ Lastly, we did not evaluate different MFI values for DSAs as the goal of our study was to determine the utility of DSA with pAMR testing using prespecified MFI cutoffs. MFI measurements have also been known to vary among HLA laboratories, limiting the transferability of MFI findings.^43^

## Conclusions

Our study findings show that pAMR testing may add prognostic value in risk-stratifying DSA+ but not DSA− patients. Future randomized controlled trials are needed to evaluate the strategy of DSA testing as the primary surveillance method, with surveillance pAMR testing reserved for DSA+ results. Additionally, moderate or severe PGD is a potential novel risk factor for dnDSAs, and earlier DSA testing at 10 to 14 days post-HTx should be considered in these patients.

## Supplementary Material

Supplementary Material

Appendix A. Supplementary data

Supplementary data associated with this article can be found in the online version at doi:10.1016/j.healun.2025.01.019.

## Figures and Tables

**Table 1:** Patient Characteristics and Transplant Outcomes by pAMR/DSA Groups

Characteristics	pAMR+/DSA+ group 1 (*n* = 45)	pAMR+/DSA− group 2 (*n* = 30)	pAMR−/DSA+ group 3 (*n* = 95)	pAMR−/DSA− group 4 (*n* = 374)	*p*-value
Donor characteristics					
Age, y, mean (SD)	32.1 (10.7)	36.6 (11.9)	31.6 (10.3)	33.0 (10.6)	0.996
Female, N (%)	12 (28.6)	5 (17.2)	20 (21.3)	54 (15.3)	0.123
Recipient characteristics					
Age, y, mean (SD)	45.7 (18.5)	58.0 (11.5)	51.0 (15.3)	55.3 (13.2)	**< 0.001**
Female, N (%)	15 (33.3)	6 (20.0)	16 (16.8)	71 (19.0)	0.136
Race					0.428
Asian, N (%)	1 (2.2)	3 (10.0)	6 (6.3)	27 (7.2)	-
Black, N (%)	8 (17.8)	5 (16.7)	14 (14.7)	42 (11.2)	-
Native American, N (%)	0	0	2 (2.1)	2 (0.5)	-
Other Race, N (%)	4 (8.9)	0	2 (7.4)	24 (6.4)	-
Pacific Islander, N (%)	0	1 (3.3)	3 (3.2)	8 (2.1)	-
White, N (%)	32 (71.1)	21 (70.0)	63 (66.3)	271 (72.5)	-
Ethnicity					
Hispanic or Latino, N (%)	17 (37.8)	7 (23.3)	34 (35.8)	107 (28.6)	0.293
Recipient BMI, mean (SD)	25.4 (4.6)	25.8 (5.1)	26.2 (4.5)	26.8 (4.3)	**0.020**
Indication for transplant					0.192
NICM, N (%)	27 (60.0)	21 (70.0)	57 (60.0)	220 (58.8)	-
ICM, N (%)	13 (28.9)	7 (23.3)	29 (30.5)	136 (36.4)	-
Congenital, N (%)	4 (8.9)	0	6 (6.3)	12 (3.2)	
Cardiac allograft failure, N (%)	1 (2.2)	2 (6.7)	3 (3.2)	6 (1.6)	-
Allosensitization pre-HTx (PRA ≥ 10%), N (%)	10 (28.6)	3 (11.5)	21 (23.9)	51 (16.6)	0.135
Durable MCS, N (%)	12 (26.7)	10 (33.3)	33 (34.7)	133 (35.7)	0.710
Medical nonadherence, N (%)	21 (46.7)	5 (16.7)	17 (17.9)	46 (12.3)	**< 0.001**
Transplant characteristics					
Multiorgan transplant, N (%)	8 (17.8)	2 (6.7)	18 (18.9)	48 (12.8)	0.245
Cold ischemic time, min, mean (SD)	195.5 (49.4)	208.4 (55.2)	200.5 (58.4)	200.1 (67.7)	0.941
Sex mismatch (female D-male R), N (%)	3 (7.1)	1 (3.4)	10 (10.6)	28 (7.9)	0.675
PHM difference, % recipient PHM, mean (SD)	7.9 (22.1)	2.8 (26.3)	4.6 (18.9)	5.2 (20.9)	0.741
Induction therapy, N (%)	24 (60.0)	17 (58.6)	46 (48.9)	169 (47.5)	0.348
DCD, N (%)	3 (6.7)	4 (13.3)	12 (12.6)	63 (16.8)	0.290
CMV mismatch (D+/R−), N (%)	7 (16.3)	6 (20.0)	20 (21.3)	72 (19.8)	0.856
Transplant outcomes					
De novo DSA, N (%)	43 (97.7)	-	86 (95.6)	-	1.000
Mixed ACR and AMR, N (%)	5 (11.1)	3 (10.0)	-	-	1.000
Cardiac allograft dysfunction, N (%)	22 (48.9)	3 (10.0)	15 (15.8)	40 (10.7)	**< 0.001**
Cardiac allograft vasculopathy, N (%)	8 (20.0)	3 (11.1)	6 (7.0)	16 (5.0)	**0.008**
Future ACR, N (%)	12 (28.6)	5 (16.7)	12 (12.8)	40 (11.1)	**0.024**

Abbreviations: ACR, acute cellular rejection; AMR, antibody-mediated rejection; BMI, body mass index; CMV, cytomegalovirus; DCD, donation after cardiac death; DSA, donor-specific antibodies; HTx, heart transplantation; ICM, ischemic cardiomyopathy; MCS, mechanical circulatory support; NICM, nonischemic cardiomyopathy; PHM, predicted heart mass; PRA, panel reactive antibodies.

Bold values indicate significant value of (p < 0.05).

**Table 2:** Single Predictor and Multipredictor Cox Proportional Hazards Analyses for Cardiac Allograft Dysfunction

Predictor	No. of patients (Total *n* = 544)	No. of events (Total *n* = 80)	HR	95% CI	*p*-value
Single predictor analysis					
Ischemic cardiomyopathy as HTx indication (vs nonischemic cardiomyopathy)	544	80	2.03	1.29–3.19	**0.002**
Allosensitization pre-HTx^a^	457	67	0.89	0.48–1.67	0.715
Durable MCS at time of HTx (yes vs no)	543	80	1.44	0.92–2.24	0.107
Medical nonadherence (yes vs no)	544	80	2.64	1.67–4.18	**< 0.001**
Donor age (by 10 y)	520	78	0.88	0.71–1.08	0.207
Induction therapy (yes vs no)	519	77	0.84	0.53–1.33	0.458
Cold ischemic time (per hour)	519	78	0.90	0.73–1.11	0.330
PHM difference (per % recipient PHM increment)	513	77	1.01	1.00–1.02	0.130
Donation after cardiac death (vs brain death)	544	80	1.37	0.69–2.71	0.373
ECMO pre-HTx (yes vs no)	540	79	2.62	0.82–8.32	0.103
pMCS pre-HTx (yes vs no)	540	79	0.55	0.28–1.11	0.096
Moderate or severe primary graft dysfunction (yes vs no)	536	78	2.09	1.25–3.49	**0.005**
De novo DSAs (vs no DSA)	528	73	2.17	1.35–3.46	**0.001**
Class I de novo DSAs alone (vs no DSA)	528	73	0.40	0.06–2.91	0.366
Class II de novo DSAs alone (vs no DSA)	528	73	1.41	0.74–2.69	0.292
Both class I and II de novo DSAs (vs no DSA)	528	73	5.91	3.36–10.40	**< 0.001**
Concurrent ACR > 1R with pAMR+ diagnosis (vs pAMR+ with ACR grade 0R)	75	25	0.66	0.15–2.81	0.574
CMV D+/R− status (vs CMV D−/R−)	531	80	1.80	0.71–4.53	0.214
Sex mismatch (female D-male R vs male D-male R)	519	78	0.58	0.21–1.58	0.285
pAMR/DSA group (vs pAMR−/DSA− group)	544	80	-	-	**< 0.001**
pAMR+/DSA+	45	22	4.35	2.58–7.35	**< 0.001**
pAMR+/DSA−	30	3	0.76	0.23–2.46	0.645
pAMR−/DSA+	95	15	1.36	0.75–2.45	0.316
pAMR−/DSA−	374	40	-	-	-
Cardiac allograft vasculopathy (vs CAV grades 0 or 1)	464	63	1.02	0.37–2.80	0.978
History of ACR > 1R (vs ACR grades 0R/1R)	530	80	1.39	0.94–2.07	0.101
Multipredictor analysis					
pAMR+/DSA+ group^b^	506	75	3.00	1.66–5.42	**0.003**
Medical nonadherence	506	75	2.18	1.31–3.62	**0.003**
Ischemic cardiomyopathy as HTx indication^c^	506	75	1.89	1.17–3.06	**0.009**
ECMO pre-HTx	506	75	5.43	1.41–20.93	**0.014**
Moderate or severe primary graft dysfunction	506	75	1.84	1.08–3.14	**0.024**
History of ACR > 1R	506	75	1.44	0.96–2.16	0.075
PHM difference	506	75	1.01	1.00–1.02	0.116
pMCS pre-HTx	506	75	0.57	0.27–1.19	0.132

Abbreviations: ACR, acute cellular rejection; CI, confidence interval; cPRA, calculated panel reactive antibodies; DSA, donor-specific antibodies; ECMO, extracorporeal membrane oxygenation; HR, hazard ratio; HTx, heart transplantation; MCS, mechanical circulatory support; pAMR, pathologic antibody-mediated rejection; PHM, predicted heart mass; pMCS, percutaneous mechanical circulatory support; UNOS, United Network for Organ Sharing.

Single predictor parameters with a *p-value < 0.15 are displayed in addition to certain clinical parameters of interest*.

Bold values indicate significant value of (p < 0.05).

a Allosensitized patients defined as having a UNOS cPRA > 10%.

b Reference is pAMR−/DSA− group.

c Reference is nonischemic cardiomyopathy.

**Table 3:** Single Predictor and Multipredictor Cox Proportional Hazards Analyses for All-Cause Death or Cardiac Retransplant

Predictor	No. of patients (Total *n* = 544)	No. of events (Total *n* = 61)	HR	95% CI	*p*-value
Single predictor analysis					
Recipient age (by 10 y)	544	61	1.02	0.86–1.21	0.793
Recipient race and ethnicity (vs non-Hispanic White)	544	61	-	-	**0.027**
Asian	37	2	0.58	0.14–2.48	0.461
Black	69	13	2.01	1.00–4.05	**0.050**
Hispanic, White	165	18	1.06	0.56–2.02	0.850
Native American	4	0	-	-	0.995
Non-Hispanic White	222	20	-	-	-
Other	35	3	1.43	0.42–4.83	0.567
Pacific Islander	12	5	6.13	2.29–16.43	**< 0.001**
Multiorgan transplant (yes vs no)	544	61	0.62	0.25–1.55	0.304
Ischemic cardiomyopathy as HTx indication (vs nonischemic cardiomyopathy)	544	61	1.55	0.20–3.55	0.107
Allosensitization pre-HTx^a^	457	44	0.98	0.47–2.05	0.962
Durable MCS at time of HTx (yes vs no)	543	61	1.13	0.68–1.89	0.639
Medical nonadherence (yes vs no)	544	61	2.33	1.37–3.96	**0.002**
Donor age (by 10 y)	520	58	0.97	0.77–1.23	0.814
Induction therapy (yes vs no)	519	58	1.23	0.71–2.11	0.463
Cold ischemic time (per hour)	519	58	0.86	0.66–1.11	0.238
PHM difference (per % recipient PHM increment)	513	58	1.00	0.99–1.01	0.775
Donation after cardiac death (vs brain death)	544	61	1.16	0.45–3.01	0.759
ECMO pre-HTx (yes vs no)	540	60	2.34	0.57–9.63	0.237
pMCS pre-HTx (yes vs no)	540	60	1.37	0.72–2.61	0.337
Moderate or severe primary graft dysfunction (yes vs no)	536	60	1.41	0.73–2.72	0.313
De novo DSAs (vs no DSAs)	533	59	1.19	0.67–2.10	0.550
Class I de novo DSAs alone (vs no DSAs)	533	59	-	-	0.996
Class II de novo DSAs alone (vs no DSAs)	533	59	0.95	0.45–2.03	0.897
Both class I and II de novo DSAs (vs no DSAs)	533	59	2.64	1.28–5.44	**0.009**
CMV D+R− status (vs D−/R−)	531	60	0.96	0.42–2.18	0.925
Sex mismatch (female D-male R vs male D-male R)	519	58	2.27	1.16–4.42	**0.016**
pAMR/DSA group (vs pAMR−/DSA− group)	544	61	-	-	**0.018**
pAMR+/DSA+	45	13	2.46	1.30–4.64	**0.006**
pAMR+/DSA−	30	4	1.08	0.38–3.03	0.887
pAMR−/DSA+	95	6	0.56	0.24–1.34	0.193
pAMR−/DSA−	374	38	-	-	-
Cardiac allograft vasculopathy (vs CAV grades 0 or 1)	474	41	2.48	1.17–5.24	**0.018**
Cardiac allograft dysfunction (yes vs no)	544	61	2.74	1.61–4.66	**< 0.001**
History of ACR > 1R (vs ACR grades 0R/1R)	530	61	0.95	0.61–1.50	0.832
Multipredictor analysis					
Cardiac allograft dysfunction	519	58	2.56	1.41–4.67	**0.002**
Recipient race and ethnicity (vs non-Hispanic White)	519	58	-	-	**0.020**
Asian	34	2	0.75	0.17–3.25	0.700
Black	68	13	1.97	0.94–4.15	0.074
Hispanic, White	159	16	0.94	0.47–1.84	0.846
Native American	4	0	-	-	0.995
Non-Hispanic White	208	19	-	-	-
Other	34	3	1.48	0.43–5.06	0.531
Pacific Islander	12	5	6.88	2.50–18.88	**< 0.001**
Sex mismatch (female D-male R vs male D-male R)	519	58	2.48	1.23–4.98	**0.011**
Medical nonadherence	519	58	1.65	0.91–3.01	0.101

Abbreviations: ACR, acute cellular rejection; CI, confidence interval; CMV, cytomegalovirus; cPRA, calculated panel reactive antibodies; DSA, donor-specific antibodies; ECMO, extracorporeal membrane oxygenation; HTx, heart transplantation; HR, hazard ratio; MCS, mechanical circulatory support; pAMR, pathologic antibody-mediated rejection; PHM, predicted heart mass; pMCS, percutaneous mechanical circulatory support; UNOS, United Network for Organ Sharing.

Single predictor parameters with a *p-value < 0.15 are displayed in addition to certain clinical parameters of interest*.

Bold values indicate significant value of (p < 0.05).

a Allosensitized patients defined as having a UNOS cPRA > 10%.

**Table 4:** Single Predictor and Multipredictor Cox Proportional Hazards Analyses for De Novo Donor-Specific Antibody Positivity

Predictor	No. of patients (Total *n* = 544)	No. of events (Total *n* = 127)	HR	95% CI	*p*-value
Single predictor analysis					
Recipient age (by 10 y)	544	127	0.84	0.75–0.94	**0.002**
Recipient race and ethnicity (vs non-Hispanic White)	544	127	-	-	0.177
Asian	37	7	1.02	0.46–2.28	0.959
Black	69	21	1.74	1.02–2.95	**0.041**
Hispanic, White	165	45	1.51	0.98–2.32	0.060
Native American	4	2	3.06	0.74–12.70	0.123
Non-Hispanic White	222	39	-	-	-
Other	35	10	2.08	1.04–4.17	**0.039**
Pacific Islander	12	3	1.51	0.47–4.90	0.489
Multiorgan transplant (yes vs no)	544	127	1.57	1.00–2.46	0.052
Ischemic cardiomyopathy as HTx indication (vs nonischemic cardiomyopathy)	544	127	0.94	0.64–1.39	0.761
Allosensitization pre-HTx^a^	457	117	1.48	0.97–2.26	0.072
Blood type (vs blood type O)	525	127	-	-	**0.007**
Blood type A	185	49	1.17	0.80–1.72	0.412
Blood type AB	22	3	0.58	0.18–1.84	0.352
Blood type B	73	19	1.24	0.74–2.08	0.423
Blood type O	245	56	-	-	-
Durable MCS at time of HTx (yes vs no)	543	127	0.85	0.58–1.22	0.371
Medical nonadherence (yes vs no)	544	127	1.88	1.28–2.77	**0.001**
Donor age (by 10 y)	520	125	0.89	0.76–1.05	0.158
Induction therapy (yes vs no)	519	124	0.96	0.67–1.37	0.826
Cold ischemic time (per hour)	519	124	1.03	0.88–1.22	0.701
Donation after cardiac death (vs brain death)	544	127	0.94	0.54–1.66	0.840
ECMO pre-HTx (yes vs no)	540	125	2.42	0.89–6.57	0.082
pMCS pre-HTx (yes vs no)	540	125	1.08	0.70–1.67	0.731
ECMO post-HTx (yes vs no)	540	125	1.53	0.67–3.49	0.307
pMCS post-HTx (yes vs no)	540	125	1.20	0.72–1.97	0.485
Moderate or severe primary graft dysfunction (yes vs no)	536	124	1.41	0.91–2.19	0.129
CMV D+/R− status (vs D−/R−)	531	127	0.75	0.41–1.38	0.354
Sex mismatch (female D-male R vs male D-male R)	519	125	1.22	0.67–2.22	0.515
History of ACR > 1R (vs ACR grades 0R/1R)	530	127	1.22	0.77–1.91	0.396
Multipredictor analysis					
Recipient age	532	122	0.84	0.75–0.95	**0.004**
Medical nonadherence	532	122	1.84	1.23–2.73	**0.003**
Moderate or severe primary graft dysfunction	532	122	1.49	0.96–2.32	0.078
ECMO pre-HTx	532	122	2.32	0.85–6.34	0.102
Multiorgan transplant	532	122	1.43	0.89–2.29	0.140

Abbreviations: ACR, acute cellular rejection; CI, confidence interval; CMV, cytomegalovirus; cPRA, calculated panel reactive antibodies; DSA, donor-specific antibodies; ECMO, extracorporeal membrane oxygenation; HTx, heart transplantation; HR, hazard ratio; MCS, mechanical circulatory support; pAMR, pathologic antibody-mediated rejection; pMCS, percutaneous mechanical circulatory support; UNOS, United Network for Organ Sharing.

Single predictor parameters with a *p-value < 0.15 are displayed in addition to certain clinical parameters of interest*.

Bold values indicate significant value of (p < 0.05).

a Allosensitized patients defined as having a UNOS cPRA > 10%.
