# Supplementary material for: Surveillance donor-specific antibody and pathologic antibody-mediated rejection testing in heart transplant patients in the contemporary era
Source: J Heart Lung Transplant. Author manuscript; Available in PMC 2025 Jul 1. (PMC12206481; doi:10.1016/j.healun.2025.01.019)
Supplement: Supplementary Material [file NIHMS2091933-supplement-Supplementary_Material.docx]

**Table S1**. Baseline characteristics of heart transplant patients and outcomes for the total study cohort. ACR, acute cellular rejection; AMR, antibody mediated rejection; BMI, body mass index; CMV, cytomegalovirus; DCD, donation after cardiac death; DSA, donor-specific antibodies; HTx, heart transplantation; ICM, ischemic cardiomyopathy; ISHLT, International Society for Heart and Lung Transplantation; MCS, mechanical circulatory support; NICM, nonischemic cardiomyopathy; pAMR, pathologic antibody mediated rejection; PHM, predicted heart mass; PRA, panel reactive antibodies.

| **Characteristics** | **No. of patients**  **(n = 544)** | **Study cohort** |
| --- | --- | --- |
| **Donor characteristics** | | |
| Age, y, mean (SD) | 520 | 32.8 (10.6) |
| Male, N (%) | 519 | 428 (82.5) |
| **Recipient characteristics** | | |
| Age, y, mean (SD) | 544 | 53.9 (14.3) |
| Male, N (%) | 544 | 436 (80.1) |
| **Race** |  |  |
| Asian, N (%) | 544 | 37 (6.8) |
| Black, N (%) | 544 | 69 (12.7) |
| Native American, N (%) | 544 | 4 (0.7) |
| Other Race, N (%) | 544 | 35 (6.4) |
| Pacific Islander, N (%) | 544 | 12 (2.2) |
| White, N (%) | 544 | 387 (71.1) |
| **Ethnicity** |  |  |
| Hispanic or Latino, N (%) | 544 | 165 (30.3) |
| Recipient BMI, mean (SD) | 521 | 26.5 (4.4) |
| **Indication for HTx** |  |  |
| NICM, N (%) | 544 | 325 (59.7) |
| ICM, N (%) | 544 | 185 (34.0) |
| Congenital, N (%) | 544 | 22 (4.0) |
| Cardiac allograft failure, N  (%) | 544 | 12 (2.2) |
| Allosensitization pre-HTx (PRA >  10%), N (%) | 457 | 85 (18.6) |
| Durable MCS, N (%) | 543 | 188 (34.6) |
| **HTx characteristics** | | |
| Multiorgan transplant, N (%) | 544 | 76 (14.0) |
| Cold ischemic time, min,  mean (SD) | 520 | 200.3 (64.0) |
| Sex mismatch (female  D-male R), N (%) | 519 | 42 (8.1) |
| PHM difference, % recipient  PHM, mean (SD) | 513 | 5.2 (20.9) |
| Induction therapy, N (%) | 519 | 256 (49.3) |
| DCD, N (%) | 544 | 82 (15.1) |
| CMV mismatch (D+/R-), N  (%) | 531 | 105 (19.8) |
| **HTx outcomes** | | |
| Moderate or severe primary graft  dysfunction, N (%) | 536 | 94 (17.5) |
| History of pAMR positivity, N (%) | 544 | 75 (13.8) |
| pAMR1i, N (%) | 75 | 44 (58.7) |
| pAMR1h, N (%) | 75 | 5 (6.7) |
| pAMR2, N (%) | 75 | 26 (34.7) |
| Mixed ACR (ISHLT grade > 2R) and  pAMR, N (%) | 75 | 8 (10.7) |
| History of DSA positivity, N (%) | 544 | 140 (25.7) |
| De novo DSA, N (%) | 134 | 129 (96.3) |
| Class I de novo DSAs alone, N (%) | 129 | 23 (17.8) |
| Class II de novo DSAs alone, N (%) | 129 | 75 (58.1) |
| Both class I and II de novo DSAs, N (%) | 129 | 31 (24.0) |
| Concurrent cardiac allograft  vasculopathy with initial pAMR positivity,  N (%) | 20 | 5 (25.0) |
| Concurrent cardiac allograft dysfunction  with initial pAMR positivity, N (%) | 72 | 17 (23.6) |

**Table S2.** Causes of death compared across pAMR/DSA groups. DSA, donor-specific antibody; pAMR, pathologic antibody mediated rejection.

| **Outcomes** | **pAMR+/DSA+**  **(n = 45)** | **pAMR+/DSA-**  **(n = 30)** | **pAMR-/DSA+**  **(n = 95)** | **pAMR-/DSA-**  **(n = 374)** | **Total** |
| --- | --- | --- | --- | --- | --- |
| All-cause mortality or cardiac retransplant (%) | 13 (28.9) | 4 (13.3) | 6 (6.3) | 38 (10.2) | 61 (11.2) |
| Cardiovascular related death (%) | 7 (15.6) | 1 (3.3) | 3 (3.2) | 6 (1.6) | 17 (3.1) |
| Infectious related mortality (%) | 1 (2.2) | 1 (3.3) | 2 (2.1) | 19 (5.1) | 23 (4.2) |
| Cancer (%) | 0 | 0 | 0 | 5 (1.3) | 5 (0.9) |
| Other cause of death (%) | 2 (4.4) | 1 (3.3) | 1 (1.1) | 4 (1.1) | 8 (1.5) |
| Unknown cause of death (%) | 1 (2.2) | 1 (3.3) | 0 | 2 (0.5) | 4 (0.7) |

**Table S3**. Cox proportional hazards analysis performed with pAMR/DSA class within the same patient as a time-dependent covariate for overall survival. CI, confidence interval; DSA, donor-specific antibody; HR, hazard ratio; pAMR, pathologic antibody mediated rejection. ^#^, reference is pAMR-/DSA- group.

| **Predictor** | **No. of pAMR/DSA classes**  **(Total n = 695)** | | | **No. of events**  **(Total n = 61)** | **HR** | **95% CI** | **p_c_-value** |
| --- | --- | --- | --- | --- | --- | --- | --- |
| pAMR+/DSA+ class^#^ | | 45 | 13 | | 4.70 | 2.37-9.29 | **<0.001** |
| pAMR+/DSA- class^#^ | | 35 | 4 | | 1.07 | 0.38-3.07 | 1.000 |
| pAMR-/DSA+ class^#^ | | 106 | 6 | | 0.99 | 0.42-2.37 | 1.000 |
| pAMR-/DSA- class | | 509 | 38 | | - | - | - |

**Table S4**. Cox proportional hazards analysis performed with pAMR/DSA class within the same patient as a time-dependent covariate for cardiac survival. CI, confidence interval; DSA, donor-specific antibody; HR, hazard ratio; pAMR, pathologic antibody mediated rejection. ^#^, reference is pAMR-/DSA- group.

| **Predictor** | **No. of pAMR/DSA classes**  **(Total n = 695)** | **No. of events**  **(Total n = 21)** | **HR** | **95% CI** | **p_c_-value** |
| --- | --- | --- | --- | --- | --- |
| pAMR+/DSA+ class^#^ | 45 | 9 | 9.83 | 3.21-30.16 | **<0.001** |
| pAMR+/DSA- class^#^ | 35 | 1 | 1.24 | 0.15-10.15 | 0.842 |
| pAMR-/DSA+ class^#^ | 106 | 3 | 2.10 | 0.54-8.10 | 0.562 |
| pAMR-/DSA- class | 509 | 8 | - | - | - |

**Table S5**. Comparison of different de novo DSA patterns for diagnosis of pathologic antibody mediated rejection. Bootstrapping, repeated 10,000 times, was implemented to generate mean and 95% confidence intervals for the positive predictive value. The p-value of the comparison of positive predictive values between DSA groups is based on the tail probability of the value of 0 (no difference) in the bootstrap distribution of the difference statistic, multiplied by two (two-tailed test). 95% confidence intervals are in parentheses. ^#^, reference is all other DSAs group. DSA, donor-specific antibody.

| **De novo DSAs** | **Positive predictive value** | **p_c_-value^#^** | **Odds ratio** | **p_c_-value^#^** |
| --- | --- | --- | --- | --- |
| Both class I and II DSAs on initial DSA+ testing | 64.2% (36.4%-88.9%) | **0.031** | 6.44 (1.14-45.82) | **0.016** |
| Progression from one DSA to both class I and II DSAs | 58.2% (28.6%-87.5%) | 0.124 | 5.02 (0.81-35.88) | 0.087 |
| Two or more class II DSAs on initial DSA+ testing | 52.9% (28.6%-77.3%) | 0.103 | 4.05 (0.85-20.26) | 0.087 |
| All other DSAs | 21.5% (12.9%-30.5%) | - | - | - |

**Table S6**. Single predictor and multipredictor Cox proportional hazards analyses for cardiac death or retransplant. Single predictor parameters with a p-value < 0.15 are displayed in addition to certain clinical parameters of interest. ACR, acute cellular rejection; CI, confidence interval; cPRA, calculated panel reactive antibodies; DSA, donor-specific antibodies; ECMO, extracorporeal membrane oxygenation; HTx, heart transplantation; HR, hazard ratio; MCS, mechanical circulatory support; pAMR, pathologic antibody mediated rejection; PHM, predicted heart mass; pMCS, percutaneous mechanical circulatory support; UNOS, United Network for Organ Sharing. *, allosensitized patients defined as having a UNOS cPRA > 10%.

| **Predictors** | **No. of patients**  **(Total n = 544)** | **No. of events**  **(Total n = 21)** | **HR** | **95% CI** | **p-value** |
| --- | --- | --- | --- | --- | --- |
| **Single predictor analysis** | | | | | |
| Recipient age (by  10-yr) | 544 | 21 | 0.73 | 0.57-0.93 | **0.010** |
| Recipient female sex  (vs. recipient male  sex) | 544 | 21 | 1.48 | 0.57-3.82 | 0.423 |
| Recipient race and  ethnicity (vs.  non-Hispanic White) | 544 | 21 | - | - | 0.222 |
| Asian | 37 | 1 | 0.88 | 0.11-7.18 | 0.906 |
| Black | 69 | 3 | 1.19 | 0.31-4.59 | 0.804 |
| Hispanic, White | 165 | 6 | 0.96 | 0.32-2.86 | 0.941 |
| Native American | 4 | 0 | - | - | 0.998 |
| Non-Hispanic White | 222 | 7 | - | - | - |
| Other | 35 | 1 | 1.61 | 0.20-13.19 | 0.658 |
| Pacific Islander | 12 | 3 | 9.82 | 2.50-38.54 | **0.001** |
| Multiorgan  transplant (yes vs.  no) | 544 | 21 | 0.79 | 0.18-3.43 | 0.757 |
| Ischemic  cardiomyopathy as  HTx indication (vs.  non-ischemic  cardiomyopathy) | 544 | 21 | 1.67 | 0.66-4.18 | 0.277 |
| Allosensitization  pre-HTx* | 457 | 16 | 1.19 | 0.38-3.68 | 0.769 |
| Durable MCS at  time of HTx (yes vs.  no) | 543 | 21 | 1.58 | 0.67-3.73 | 0.295 |
| Medical  nonadherence (yes  vs. no) | 544 | 21 | 4.15 | 1.75-9.82 | **0.001** |
| Donor age (by 10-yr) | 520 | 19 | 1.30 | 0.88-1.93 | 0.188 |
| Induction therapy (yes  vs. no) | 519 | 19 | 1.22 | 0.48-3.13 | 0.675 |
| Cold ischemic time  (per hour) | 519 | 19 | 0.84 | 0.53-1.33 | 0.454 |
| PHM difference  (per % recipient  PHM increment) | 513 | 19 | 1.00 | 0.98-1.02 | 0.839 |
| Donation after  cardiac death (vs.  brain death) | 544 | 21 | 2.84 | 0.55-14.65 | 0.212 |
| Moderate or severe  primary graft  dysfunction (yes vs.  no) | 536 | 20 | 3.20 | 1.20-8.51 | **0.020** |
| ECMO pre-HTx (yes  vs. no) | 540 | 20 | 3.96 | 0.53-29.88 | 0.182 |
| pMCS pre-HTx (yes  vs. no) | 540 | 20 | 0.28 | 0.04-2.10 | 0.215 |
| De novo DSAs (vs. no  DSA) | 533 | 20 | 3.40 | 1.41-8.22 | **0.007** |
| Class I de  novo DSAs alone (vs.  no DSA) | 533 | 20 | - | - | 0.997 |
| Class II de  novo DSAs alone (vs.  no DSA) | 533 | 20 | 2.66 | 0.89-7.97 | 0.080 |
| Both class I and II  de novo DSAs (vs. no  DSA) | 533 | 20 | 7.16 | 2.54-20.14 | **<0.001** |
| Sex mismatch (female  D-male R vs. male  D-male R) | 519 | 19 | 1.71 | 0.49-5.93 | 0.397 |
| pAMR/DSA group (vs.  pAMR-/DSA- group) | 544 | 21 | - | - | **0.003** |
| pAMR+/DSA+ | 45 | 9 | 6.55 | 2.52-17.05 | **<0.001** |
| pAMR+/DSA- | 30 | 1 | 1.17 | 0.15-9.37 | 0.882 |
| pAMR-/DSA+ | 95 | 3 | 1.34 | 0.36-5.07 | 0.664 |
| pAMR-/DSA- | 374 | 8 | - | - | - |
| Cardiac allograft  vasculopathy (vs. CAV  grades 0 or 1) | 474 | 19 | 5.31 | 2.08-13.58 | **<0.001** |
| Cardiac allograft  dysfunction (yes vs.  no) | 544 | 21 | 6.30 | 2.65-14.98 | **<0.001** |
| History of ACR > 1R  (vs. ACR grades  0R/1R) | 530 | 21 | 2.47 | 1.00-6.13 | **0.051** |
| **Multipredictor analysis** | | | | | |
| Cardiac allograft  dysfunction | 448 | 17 | 5.96 | 1.99-17.81 | **0.001** |
| Recipient Age | 448 | 17 | 0.69 | 0.50-0.95 | **0.025** |
| Donor Age | 448 | 17 | 1.61 | 1.01-2.58 | **0.046** |
| Medical  nonadherence | 448 | 17 | 2.73 | 0.96-7.71 | 0.059 |
| Moderate or severe  primary graft  dysfunction | 448 | 17 | 2.33 | 0.80-6.76 | 0.119 |
| Cardiac allograft  vasculopathy | 448 | 17 | 2.37 | 0.77-7.24 | 0.131 |
